# Synovial Folds Impingement After Unicondylar Knee Arthroplasty

**DOI:** 10.7759/cureus.54147

**Published:** 2024-02-13

**Authors:** Naoya Kikuchi, Yu Taniguchi, Akihiro Kanamori, Masashi Yamazaki

**Affiliations:** 1 Department of Orthopaedic Surgery, Institute of Medicine, University of Tsukuba, Tsukuba, JPN; 2 Department of Orthopaedic Surgery, Ichihara Hospital, Tsukuba, JPN

**Keywords:** clicking, synovial fold impingement, pain, painful knee, medial compartment osteoarthritis of knee

## Abstract

Soft tissue impingement after total knee arthroplasty has been reported; however, complications after unicondylar knee arthroplasty (UKA) have rarely been reported. We report a rare case of synovial fold impingement that occurred after UKA and caused severe pain with clicking during knee flexion and extension. Diagnostic arthroscopy was performed 3 weeks after UKA and found that a hypertrophied and congested synovial fold in the medial compartment impinged on the femoral component during knee flexion and extension. After excising the synovial fold, the patient’s symptoms improved. *Synovial fold impingement* is a complication that should be considered when patients complain of severe pain with clicking in the knee after UKA.

## Introduction

Unicondylar knee arthroplasty (UKA) is an established treatment for unicompartmental osteoarthritis of the knee, with reportedly good outcomes [1,2]. Despite high success rates, UKA is associated with various potential sources of postoperative pain, which surgeons must consider. These include septic or aseptic loosening, loose bodies, implant failure, and chronic regional pain syndrome [3,4]. However, instances of soft tissue impingement, particularly involving synovial folds, are exceedingly rare post-UKA. Our case report highlights the unusual occurrence of synovial fold impingement post-UKA, elucidating its diagnostic challenges and therapeutic intervention.

## Case presentation

**Figure 1:**
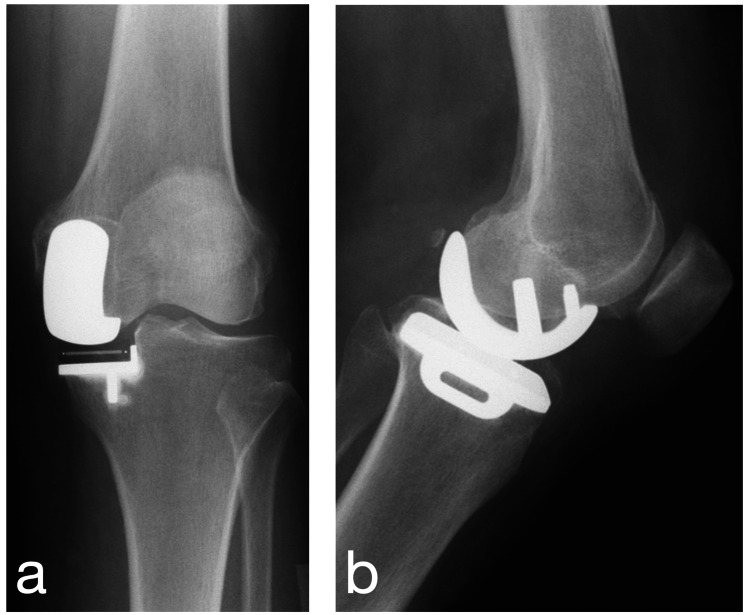
Postoperative radiographs. (a) Anteroposterior view. (b) Lateral view.

A 77-year-old woman with a 10-year history of osteoarthritis was treated with left UKA, using the mini-midvastus approach. Preoperative physical examination demonstrated that the active range of motion was 0-130°, and there was no pain at the patellofemoral joint. An Oxford® mobile-bearing medial UKA implant (Zimmer-Biomet, Warsaw, Indiana, USA) was used, with a “small” femoral component, a “AA” tibial component, and a 4-mm polyethylene insert (Figure 1). No synovial fold was found that would cause clicking during surgery.

Three days after surgery, while undergoing a range of motion exercise with a physical therapist, the patient complained of severe pain localized to the medial patellar border with clicking during knee flexion and extension. There were no problems with the implant dynamics under fluoroscopy. An infection workup showed normal results, including blood count and C-reactive protein level. 

**Figure 2:**
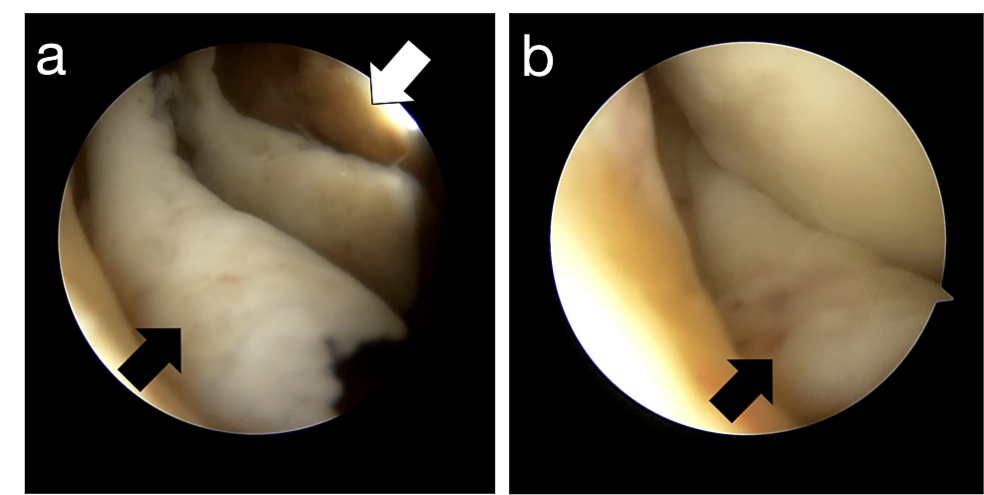
Arthroscopic findings before the procedure The hypertrophied synovial fold in the medial compartment were observed to impinge the femoral component during knee (a) flexion and (b) extension. The white arrow shows the femoral component. The black arrow shows impinged synovial fold.

**Figure 3:**
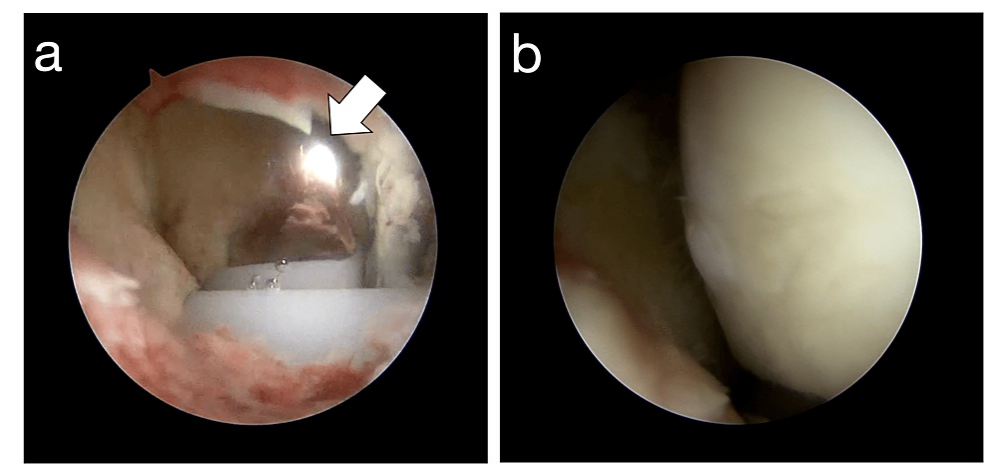
Arthroscopic findings after the procedure Synovial fold was excised to prevent impingement during knee (a) flexion and (b) extension. The white arrow shows the femoral component.

Three weeks after the primary UKA, a diagnostic arthroscopy was performed to clarify the cause of her symptoms. During the arthroscopic evaluation, it was found that hypertrophied synovial fold in the medial compartment impinged on the femoral component during knee flexion and extension (Figure 2). Synovial fold was excised using a shaver so that it no longer impinged during knee flexion and extension (Figure 3).

Postoperatively, her symptoms promptly resolved. She also regained the same range of motion as before the primary surgery. Four years postoperatively, the patient maintained the previous range of motion, and the left knee remained asymptomatic. As her symptoms improved with synovial fold resection, we can attribute these symptoms to synovial fold impingement after UKA.

## Discussion

Although UKA is a successful procedure in most patients, postoperative complications rarely occur. In a large-scale survey of complications after UKA [5], the incidence of complications was 89 knees (5.9%) after 1,576 UKA procedures, and only two cases (0.2%) of impingement were reported. For the treatment of impingement in two knees, arthroscopic bone cement and osteophyte removal were performed. 

There are two case reports of pseudomeniscal synovial impingement [6] on soft tissue impingement after UKA, similar to this case. These two cases experienced symptoms 5 months and 1 year postoperatively, demonstrated synovial cell-lined, thick fibrous tissue with degeneration, and responded well to arthroscopic excision. In our case, the symptoms were improved by arthroscopic surgery in the same way; however, the symptoms occurred immediately after the operation, and the onset time was different. After UKA, it is difficult to diagnose soft tissue impingement after UKA using MRI for artifacts; therefore, diagnostic arthroscopy should be performed when it is suspected.

Generally, the synovial folds of the knee are embryological remnants of the membranes that separate compartments. Mediopatellar synovial folds are reported in 79.9% of Japanese people [7]. Plica syndrome, which is thought to be similar to this case, is caused by an inflammatory process secondary to acute trauma, repetitive stress injury, meniscal tears, or loose bodies [8]. In this report, the synovial fold was congested and hypertrophied, which could have been caused immediately after surgery by surgical procedure. 

**Figure 4:**
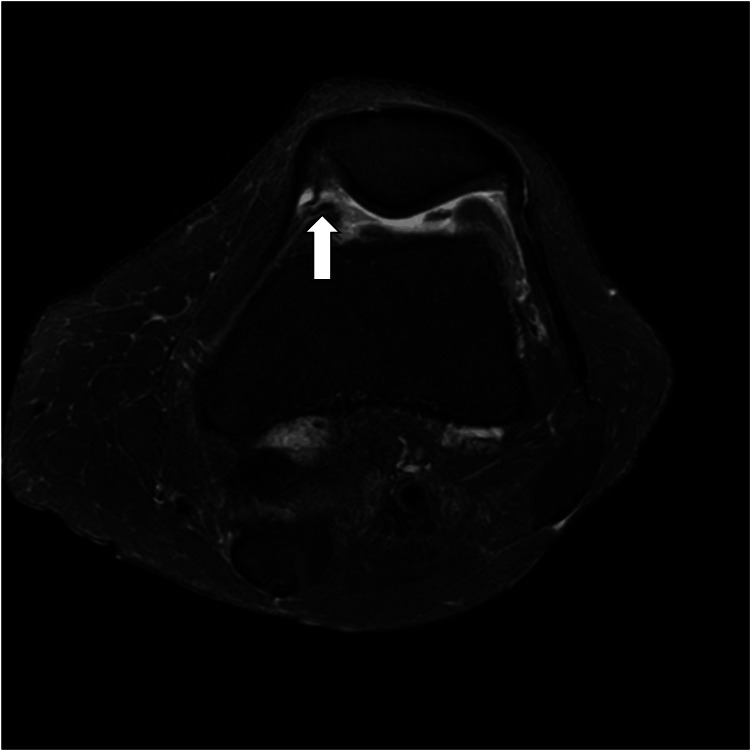
Preoperative axial fat-suppressed T2WI image The white arrow shows synovial hyperplasia in the knee joint.

Reinterpreting preoperative MRI retrospectively, synovial hyperplasia was found (Figure 4). The synovial fold was inconspicuous during the primary operation, and no synovectomy was performed. Synovial folds may cause soft tissue impingement in total knee arthroplasty. Although its incidence is very low (0.07%) [9], synovectomy is considered important. Synovectomy could also be effective in UKA to prevent soft-tissue impingement.

There is a critical limitation to this report. A histopathological examination was not performed. Considering the position of the removed soft tissue, it was not considered to be a remnant meniscus. Histopathological examination should be performed to conduct a more detailed examination.

## Conclusions

We reported a case of synovial fold impingement after UKA. Surgeons should be aware of the presence of synovial fold impingement as a postoperative complication after UKA. Arthroscopy may be useful to diagnose and treat suspected soft tissue impingement after UKA.
